# Aspergillus fumigatus Septic Arthritis of the Wrist: A Report of a Rare Case in an Immunocompromised Patient

**DOI:** 10.7759/cureus.43622

**Published:** 2023-08-17

**Authors:** Ersin Taskin, Ulas Yavuz, Derya Akbaba, Muhammed Yusuf Afacan, Mahmut Kursat Ozsahin, Gokhan Kaynak, Ali Seker

**Affiliations:** 1 Department of Orthopaedics and Traumatology, Istanbul University-Cerrahpasa, Cerrahpasa Medical Faculty, Istanbul, TUR

**Keywords:** carpectomy, non-hodgkin lymphoma (nhl), wrist septic arthritis, invasive aspergillosis, aspergillus fumigatus

## Abstract

*Aspergillus fumigatus* is a saprophytic fungus encountered as a pathogen in airborne lung infections. Although it commonly causes pulmonary infectious diseases, when disseminated, it may cause a systemic infection termed invasive aspergillosis, which is associated with high mortality and morbidity. Virtually, all organ systems may be involved. However, the musculoskeletal system is relatively uncommon. Here, we present a case of invasive aspergillosis in an immunocompromised patient involving the wrist joint, an exceedingly rare site. Our treatment choice is serial open debridement, irrigation, and intravenous antibiotics. This case study exemplifies the potential challenges in the identification and treatment of such an uncommon clinical condition. A thorough clinical and microbiological evaluation is essential for accurate diagnosis of fungal septic arthritis of the wrist. Aggressive early surgical treatment combined with appropriate early intravenous antibiotics is crucial for eradicating joint infection.

## Introduction

Septic arthritis is an orthopedic emergency that may lead to the destruction of the joint and may be fatal even in the hands of surgeons specialized in musculoskeletal diseases [1]. Septic arthritis of the wrist may be defined as the infection of the radiocarpal joint but may also involve the surrounding joints and soft tissues [2]. Although the most common joint targeted by the disease is the knee, involvement of every joint throughout the body has been reported in the literature [3]. Although septic arthritis of the wrist is an uncommon clinical condition, it requires urgent surgical intervention and antibiotic treatment. If left untreated, it may cause significant morbidity and even mortality [4].

*Aspergillus fumigatus*, a saprotrophic fungus that is ubiquitously found in the environment, is known to cause airborne infections by inhalation of conidia [5]. The infections caused by this pathogen vary depending on the immunologic status of the host and if there is a structural lesion or an inflammatory condition in the lungs [6].

Patients frequently receiving cytotoxic treatment for hematologic malignancies may develop invasive aspergillosis, a systemic fungal infection of *Aspergillus fumigatus*. Due to the angio-invasive properties of the pathogen, it may become a disseminated disease and present with extrapulmonary involvement in distinct organ systems. The musculoskeletal system can be involved but is a very occasional site for disseminated invasive aspergillosis [7].

This report demonstrates a rare case of *Aspergillus fumigatus* septic arthritis of the wrist due to invasive aspergillosis in an immunocompromised patient. This case study illustrates the potential challenges in identifying and treating such an uncommon clinical condition and shows the importance of early surgical intervention. In delayed cases, the result may be death.

## Case presentation

A 48-year-old male patient, diagnosed with non-Hodgkin lymphoma 10 months ago, was admitted to our hospital’s hematology ward due to a lymphoma relapse. The patient was consulted for pain, tenderness, swelling, and restricted motion of the right wrist. He had a history of recent chemotherapy and long-term cortisone use. Apart from his hematologic malignancy, he had no other chronic diseases.

**Figure 1:**
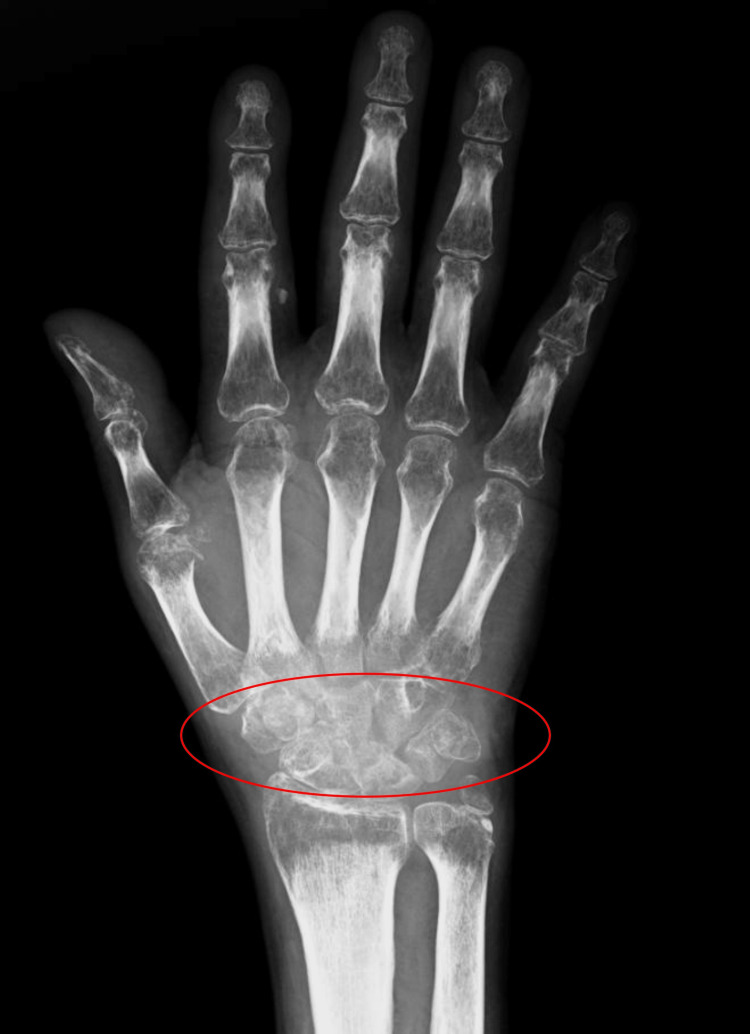
Preoperative hand anteroposterior X-ray image demonstrates osteolysis of the carpal bones. Diffuse osteolytic changes in the carpal bones in the region inside the red circle of the hand anteroposterior X-ray image are noteworthy.

His complaints about the right wrist had appeared four months ago and progressively increased in severity. His history was remarkable for multiple insertions of intravenous lines on the dorsum of the right wrist. A physical examination of the patient revealed edema, erythema, and warmth on the wrist joint. Both the active and passive range of motion of the wrist were severely restricted and painful. On the plain radiographs, destruction of carpal bones was observed (Figure 1).

**Figure 2:**
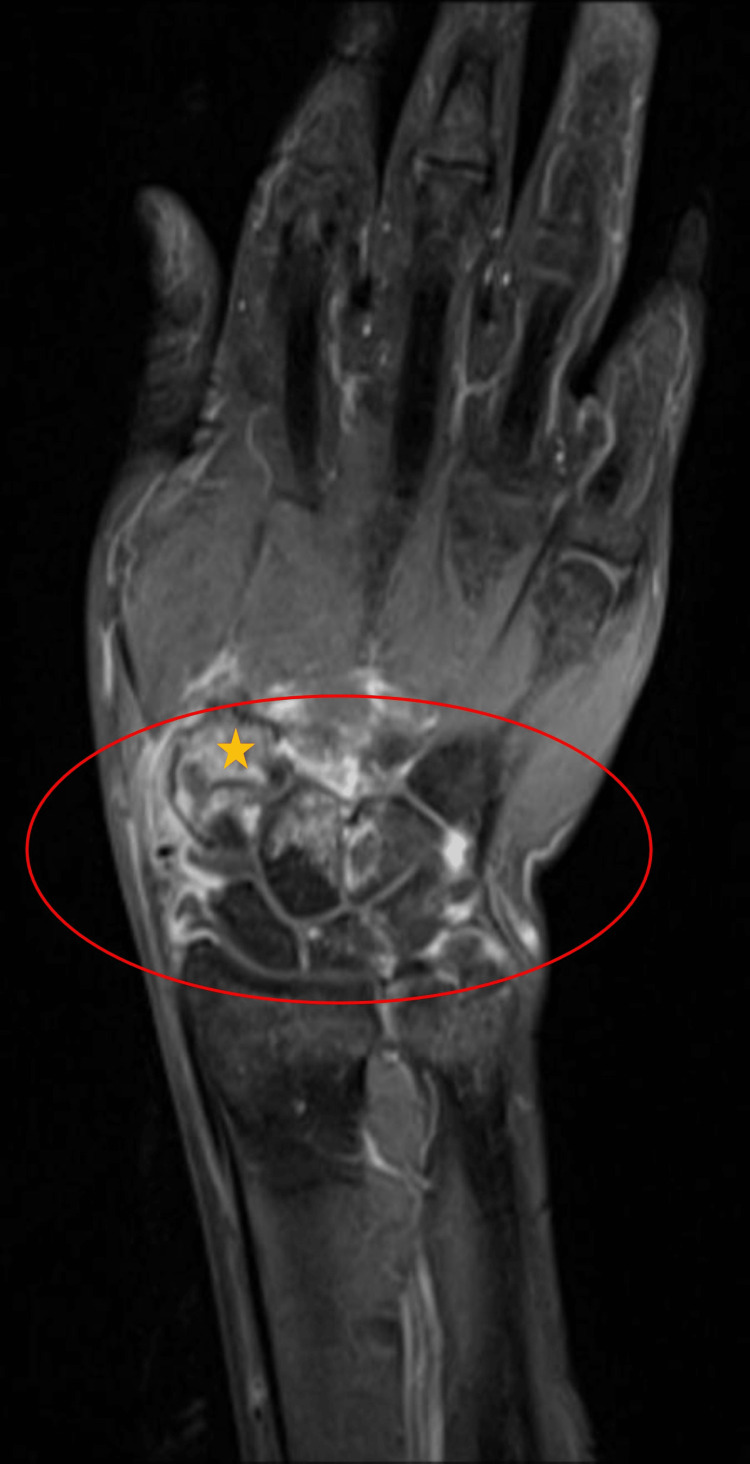
MRI demonstrates wrist joint effusion and bone marrow edema of the carpal bones. Wrist joint effusion and bone marrow edema in the carpal bones in the region inside the red circle are noteworthy. The white areas around the joint indicate the joint effusion. The orange star illustrates the bone marrow edema in the carpal bones. MRI: magnetic resonance imaging

Contrast-enhanced magnetic resonance imaging (MRI) scan demonstrated carpal joint effusion and findings consistent with osteolysis and osteomyelitis of carpal bones (Figure 2).

**Figure 3:**
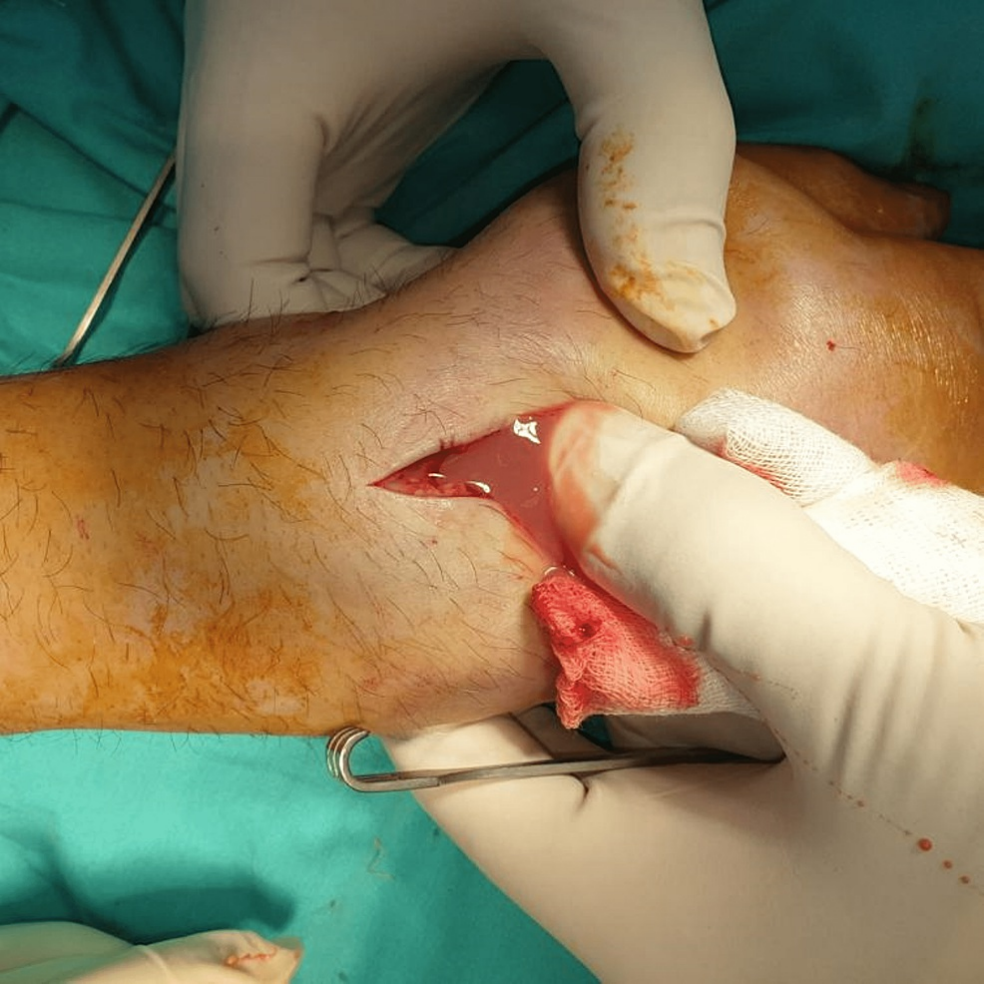
Intraoperative image during the open debridement and irrigation of the wrist joint. Purulent material was spontaneously drained out of the joint cavity following the arthrotomy.

At the time, C-reactive protein (CRP) levels were elevated, but white blood cell (WBC) count and polymorphonuclear leukocyte percentage (PMN%) were decreased due to the chemotherapy regimen. The patient was on broad-spectrum antibiotic therapy, including trimethoprim/sulfamethoxazole, cefoperazone/sulbactam, teicoplanin, tigecycline, and liposomal amphotericin B, due to very low WBC count and fever. Septic arthritis of the wrist was suspected, and the patient was taken to the operating room for open debridement and irrigation. A midline dorsal incision was used. The pus was drained from the joint cavity (Figure 3), and thorough debridement of infected tissues was performed.

**Figure 4:**
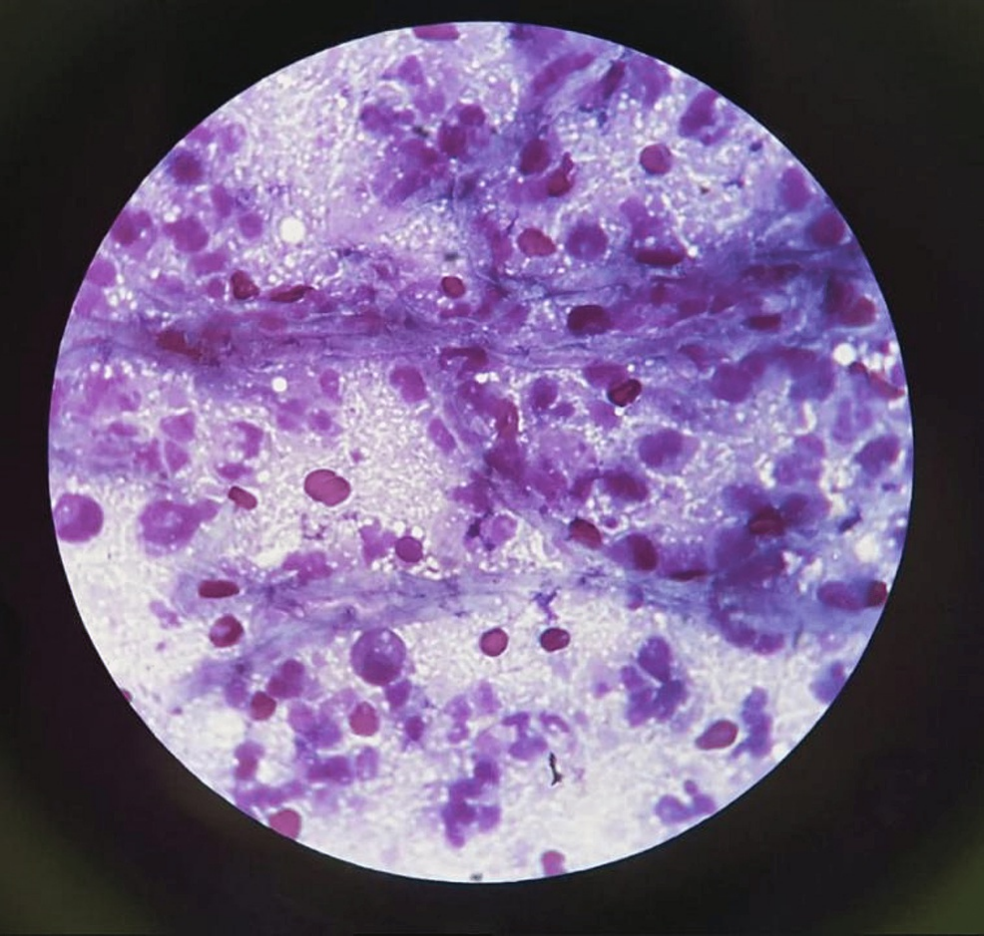
Microscopic evaluation of the joint fluid specimen. May-Grünwald-Giemsa stain showing polymorphonuclear leukocytes, synovial cells, and *Aspergillus* hyphae.

**Figure 5:**
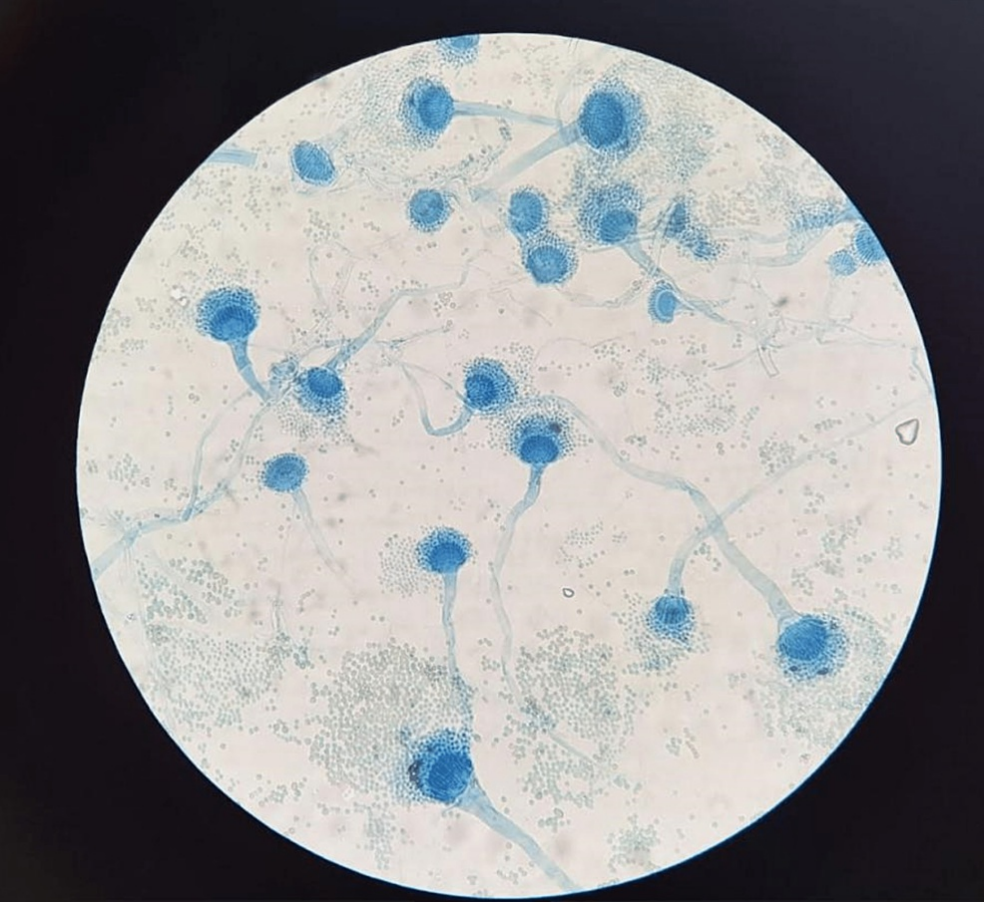
Microscopic evaluation of the joint fluid specimen. Lactophenol cotton blue stain demonstrating *Aspergillus fumigatus*.

The joint cavity was irrigated using saline. The collected specimen was sent to microbiology and pathology for evaluation (Figures 4, 5).

*Aspergillus fumigatus* was detected both on the joint fluid specimen and blood, and the antifungal therapy was begun accordingly. The regimen of choice was intravenous voriconazole. Despite the surgical and antifungal therapy, erythema, swelling, and purulent drainage from the wound persisted. A follow-up MRI scan demonstrated joint effusion with density consistent with pus.

Despite the antifungal therapy and surgical debridement of the wrist joint, purulent drainage persisted. Therefore, we considered osteomyelitis of the carpal bones to be the reason for the persistence of the infection, and the patient was taken to the operating room for a total carpectomy. The joint cavity was approached using the previous dorsal incision. All carpal bones were resected. The infected tissues including the proximal parts of the metacarpals were carefully debrided, and the joint space was irrigated using saline. An external fixator was used to achieve joint stability. Vacuum-assisted closure therapy was initiated to prevent fluid collection and accelerate wound healing.

A week after the total carpectomy, inflammatory findings on the wrist joint subsided and CRP levels regressed to the normal range. The patient was taken to the operating theater to remove the VAC system and debride the remaining wound. Seven days after the final debridement, the patient had a fever, and his condition progressively got worse. Two days later, he died from septic shock in the intensive care unit.

## Discussion

A vast majority of septic arthritis cases of the wrist, in concordance with septic arthritis in general, are caused by bacteria, with *Staphylococcus aureus* being the most common species [2]. Fungal agents are rare causes of wrist joint infections. According to a study including 40 patients diagnosed with carpal joint infections, only two cases were associated with fungi [4]. Our case demonstrates that rare infectious agents should always be considered in immunocompromised patients.

Septic arthritis of the wrist is not a common clinical entity. It constitutes 5%-23% of upper extremity septic arthritis cases [8]. Pain, tenderness, warmth, and restricted range of motion in both hand and wrist is common, but fever is present only in 18% of cases [4]. Although white blood cell counts, erythrocyte sedimentation rate, and C-reactive protein levels are elevated in septic arthritis, joint aspirate analysis is recommended for diagnosis since they are nonspecific markers of systemic inflammation [2]. However, since the wrist is a relatively small joint with a lesser amount of joint fluid, it may not be possible to obtain an adequate amount of sample even under ultrasonographic guidance. In the presented case, we were informed that before our evaluation, the patient had two attempts of ultrasound-guided joint aspiration, which both ended up as dry tap.

*Aspergillus* arthritis can be treated by surgery combined with antifungal agents [9]. The recommended first line of antifungal therapy against *Aspergillus* arthritis is intravenous voriconazole. If an adequate response to voriconazole is not observed, posaconazole can be given as the second-line treatment [10].

*Aspergillus fumigatus* is an exceedingly rare cause of septic arthritis. Although *Aspergillus* arthritis has a high therapy response rate (71%), the mortality rate is also high (35%). Therefore, these patients should be under careful surveillance. A study reviewing 31 cases of *Aspergillus* arthritis included only one case of carpal involvement. It usually spreads to the wrist joint by hematogenous seeding but may also infect the joint by direct inoculation [9]. In our case, the patient has a history of multiple intravenous catheter insertions over the wrist, which may have eased the route of spread as direct inoculation.

## Conclusions

*Aspergillus fumigatus* arthritis of the wrist is a rare condition. In immunocompromised patients, uncommon joint involvements and pathogens should be considered since delayed diagnosis and treatment of septic arthritis may be associated with significant morbidity and mortality in this population. If the patient had been operated on four months ago, he may have survived the septic arthritis. Even with a suspicion of septic arthritis, arthrocentesis shall be performed, and the fluid analysis should contain the fungi and other rarely seen organisms.
